# Optimizing Laser Capture Microdissection Protocol for Isolating Zone-Specific Cell Populations from Mandibular Condylar Cartilage

**DOI:** 10.1155/2019/5427326

**Published:** 2019-11-21

**Authors:** Aisha M. Basudan, Yanqi Yang

**Affiliations:** ^1^Division of Orthodontics, Dental Services Department, Ministry of National Guard-Health Affairs, Riyadh, Saudi Arabia; ^2^King Abdullah International Medical Research Center (KAIMRC), Riyadh, Saudi Arabia; ^3^College of Dentistry King Saud Bin Abdulaziz University for Health Sciences (KSAU-HS), Riyadh, Saudi Arabia; ^4^Department of Orthodontics, Faculty of Dentistry, The University of Hong Kong, Pokfulam, Hong Kong

## Abstract

Mandibular condylar cartilage (MCC) is a multizonal heterogeneous fibrocartilage consisting of fibrous (FZ), proliferative (PZ), mature (MZ), and hypertrophic (HZ) zones. Gross sampling of the whole tissue may conceal some important information and compromise the validity of the molecular analysis. Laser capture microdissection (LCM) technology allows isolating zonal (homogenous) cell populations and consequently generating more accurate molecular and genetic data, but the challenges during tissue preparation and microdissection procedures are to obtain acceptable tissue section morphology that allows histological identification of the desirable cell type and to minimize RNA degradation. Therefore, our aim is to optimize an LCM protocol for isolating four homogenous zone-specific cell populations from their respective MCC zones while preserving the quality of RNA recovered. MCC and FCC (femoral condylar cartilage) specimens were harvested from 5-week-old Sprague–Dawley male rats. Formalin-fixed and frozen unfixed tissue sections were prepared and compared histologically. Additional specimens were microdissected to prepare LCM samples from FCC and each MCC zone individually. Then, to evaluate LCM-RNA integrity, 3′/m ratios of glyceraldehyde 3-phosphate dehydrogenase (GAPDH) and beta-actin (*β*-Actin) using quantitative reverse transcription-polymerase chain reaction (qRT-PCR) were calculated. Both fixed and unfixed tissue sections allowed reliable identification of MCC zones. The improved morphology of the frozen sections of our protocol has extended the range of cell types to be isolated. Under the empirically set LCM parameters, four homogeneous cell populations were efficiently isolated from their respective zones. The 3′/m ratio means of GAPDH and *β*-Actin ranged between 1.11–1.56 and 1.41–2.12, respectively. These values are in line with the reported quality control requirements. The present study shows that the optimized LCM protocol could allow isolation of four homogenous zone-specific cell populations from MCC, meanwhile preserving RNA integrity to meet the high quality requirements for subsequent molecular analyses. Thereby, accurate molecular and genetic data could be generated.

## 1. Introduction

Unlike most synovial joints, articular surfaces of the temporomandibular joint are covered by fibrocartilage, and the one covering the condyle is known as mandibular condylar cartilage (MCC). Mandibular condyle dysfunctions might be associated with temporomandibular joint (TMJ) disorders such as internal derangement, osteoarthrosis, and traumas [1]. Approximately 10-11% of the people suffering from temporomandibular joint disorders have symptoms of TMJ-osteoarthrosis [2, 3]. MCC exhibits distinctive biological characteristics as compared with other types of cartilages. Functionally, MCC plays a dual role: as a growth plate cartilage and as an articular cartilage [4, 5]. Histologically, MCC is a multizonal heterogeneous fibrocartilage which is composed of fibrous (FZ), proliferative (PZ), mature (MZ), and hypertrophic (HZ) zones [1, 6].

While the reliability of molecular studies heavily relies on the procurement of homogenous cell populations [7], native tissues are inherently heterogeneous. Traditional gross sampling would eventually result in average values of different cell phenotypes, concealing important information, and probably questioning the validity of the subsequent analysis [8]. Manual microdissection [9–11] may allow zonal isolation from the tissue studied, but it is time-consuming and requires excellent manual skills. This technique is not applicable for the small-sized animals like rats and mice. In addition, isolation of several zones from MCC via this technique is not possible for its relatively smaller size, as well as its irregular zonal distribution [7]. Other nonmanual techniques have been attempted to isolate the MCC zones, such as countercurrent centrifugal elutriation [12] and fluorescence-activated cell sorting technique [2], but the former technique is not very sensitive, and the latter one requires to culture the assorted cells prior to gene expression profiling. Laser capture microdissection (LCM) technology, on the other hand, allows precise procurement of cells of interest from a heterogeneous tissue rapidly and in a practical manner [13]. Murakami et al. succeeded to use infrared (IR) LCM to procure two groups of cells: one from the superficial fibrous-like layer (FZ and PZ) and the other one from the deeper cartilage-like layer (MZ and HZ) of the MCC [5]. Capturing cells from two zones (e.g., FZ and PZ) and joining them in one group (e.g., superficial fibrous-like layer) made it impossible to discern which subpopulation of cells in the same group is the source of the data obtained in Murakami's study [14].

The use of LCM in conjunction with the various molecular analyses could clarify many hidden or masked diagnostic and therapeutic aspects which were not previously identified. Heterogeneity of procured cells has been an impediment in most of the MCC molecular studies. To date, no study has successfully isolated homogenous cell populations from their respective zones; in other words, it was not possible to isolate fibroblast-like cells from FZ, proliferative cells from PZ, mature chondrocytes from MZ, and hypertrophic chondrocytes from HZ. The aim of this study is to optimize a protocol for isolating several homogenous cell populations from their respective MCC zones of rats using the LCM technique. This protocol has successfully coped with three challenges: preserving the histomorphology of tissue sections to be microdissected, feasibility of capturing four homogenous cell populations one from each zone of MCC tissue, and minimizing RNA degradation during the different LCM-protocol procedures.

## 2. Materials and Methods

**Figure 1:**
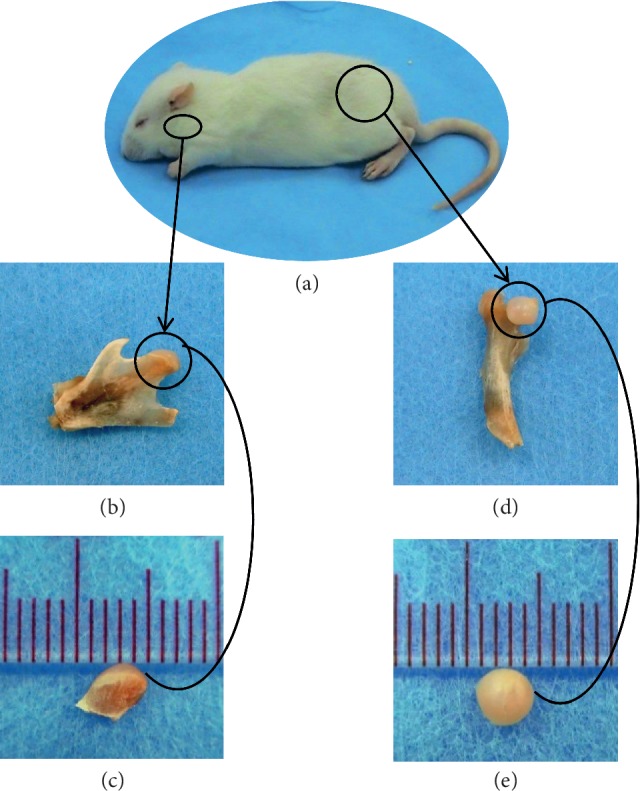
Tissue procurement from the experimental model. (a) Five-week-old male SD rats with the TMJ and femoral joint encircled. (b) Mandibular ramus dissected with the mandibular condyle encircled. (c) MCC with minimal condylar bone was harvested. (d) Femoral bone dissected with the femoral condyle encircled. (e) FCC without the underlying bone was harvested.

As numerous properties are shared, a 5-week-old male Sprague–Dawley rat (Rattus norvegicus) was chosen as an experimental model. We selected the age of 5 weeks not only because MCC articulation function is already present in a more mature state but also because the maximum growth spurt for rats occurs at day 31.5. The use of these animals was approved by the Committee on the Use of Live Animals in Teaching and Research of the University of Hong Kong (CULATR 2311-11), and the procedures were carried out in accordance with the institutional guidelines. The animals were kept under the standardized conditions at the Laboratory Animal Unit of The University of Hong Kong/the Minimal Disease Area with controlled humidity-temperature environment, controlled light-dark regime (artificial light for 12 hours daily), sufficient movement allowed, free access to water, and hygienic conditions were provided for the rats. Animals were sacrificed by intraperitoneal injection using 20% Dorminal (200 mg pentobarbital sodium, Alfasan, Woerden-Holland, Netherlands) with a dose of 100 mg per 100 g of body weight (see Supplementary Materials [Supplementary-material supplementary-material-1]). Mandibular condyles and femoral condylar cartilage (FCC) were aseptically removed (Figure 1) to analyze two primary outcomes: histology of prepared tissue sections and RNA integrity of LCM samples.

**Figure 2:**
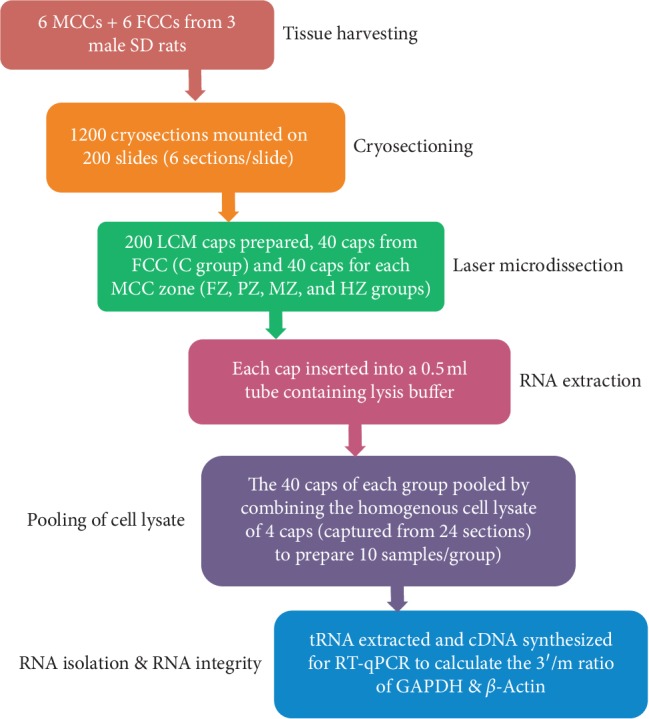
Schematic workflow for the experiment of isolating homogenous cell populations from FCC and MCC zones using the LCM technique.

For the histological comparison of formalin-fixed paraffin-embedded (FFPE) sections and unfixed frozen sections, MCCs dissected from one animal were either immediately frozen using the protocol described below or fixed in 4% formalin, decalcified, dehydrated, embedded in paraffin, sectioned, and briefly stained with cresyl violet stain. In addition, six MCC and six FCC specimens were harvested from three rats to prepare 50 LCM samples: 10 for each MCC zone (FZ, PZ, MZ, and HZ groups) and 10 samples from FCC tissue (group C) (Figure 2). Then, to evaluate the integrity of LCM-RNA, qRT-PCR was performed to determine 3′/m ratio of two housekeeping genes: GAPDH and *β*–Actin.

**Figure 3:**
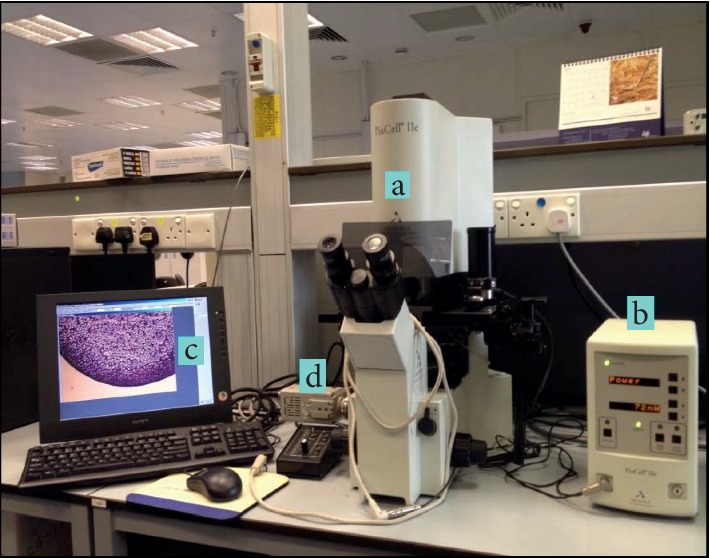
PixCell II laser capture microdissection instrument: (a) infrared laser and inverted light microscope, (b) laser control tower, (c) PC monitor with live video display, and (d) video camera.

An optimized protocol that improves the quality of RNA recovered from LCM samples of MCC zones is illustrated below in terms of specimen freezing, cryosectioning, staining and dehydration, preparation for LCM procedure, empirical setting of LCM parameters and performing laser microdissection using IR type of laser (Arcturus PixCell® II Laser Capture Microdissection System, CA, USA) (Figure 3), RNA extraction and pooling of cell lysates, RNA isolation, and evaluation of RNA integrity. All procedures were carried out under very strict RNase-free conditions (see Supplementary Materials [Supplementary-material supplementary-material-1]).

### 2.1. Specimen Freezing

Right and left MCC specimens dissected from the same animal were placed onto a precooled OCT layer (Optimal Cutting Temperature grade media, Tissue-Tek, Sakura Finetek, USA) in one cryomold. Then, OCT was added to the mold and frozen into the cooled isopentane (Sigma-Aldrich, USA), and then frozen specimens can be stored in a −70°C freezer.

### 2.2. Cryosectioning

The cryostat temperature was set to −24°C to −30°C, and the specimen stage, working surfaces, brushes (used to straighten the newly cut sections), microslide boxes and microscope slides were prepared according to the RNA-free conditions (see Supplementary Materials [Supplementary-material supplementary-material-1]). After wiping down the cryostat chamber with 100% ethanol and installing a new microtome blade, the OCT-embedded specimen was transferred from the −70°C freezer to the cryostat to equilibrate and then mounted securely to the specimen stage. To start sectioning, an empty microslide box with a small desiccant pack was placed on dry ice near the cryostat, and then excess OCT was removed by sectioning at 10–20 *μ*m, but when getting close to the specimens, the cutting thickness was set to 7 *μ*m. The cut sections were mounted onto the central area of a precleaned glass microscope slide (HistoBond®+ adhesive microscope slides, Marienfeld laboratory glassware, Germany), which were kept at room temperature. However, after mounting the first section, the slide should be kept in the cryostat till the remaining sections are cut and placed on it. This should be performed as quickly as possible to avoid RNA degradation. We mounted three cryosections per slide, which is equivalent to six tissue sections as we have embedded two specimens in one cryomold. Every tenth slide had only one section, which was then stained to provide a guide map during LCM procedures. The slides with the cryosections were placed in the chilled small-size slide box on dry ice container, which was sealed with laboratory film and stored at −70°C. Since the MCC tissue is relatively small, it is recommended to section the whole block in one setting.

### 2.3. Staining and Dehydration

Arcturus HistoGene™ LCM Frozen Section Staining Kit (CA, USA) was used according to the manufacturer's instructions, but with a few modifications:

- Tissue sections were dehydrated in absolute ethanol twice, using two 100% ethanol jars.
- Desiccant beads (4 Å beads, Sigma-Aldrich, Germany) were added to 100% ethanol and xylene jars.
- 50 *μ*L of ProtectRNA RNase inhibitor (500x concentrates, Sigma-Aldrich, USA) was added to all jars (the RNase-free water jars and ethanol dehydration series jars) except the one containing xylene.
- The first 75% ethanol jar was placed at −20°C in a freezer, while the remaining jars are kept on ice. After immersion, in the first 75% ethanol, a rinse in RNase-free water jar was applied.
- Then, the staining step and the following (poststaining) water wash were skipped for tissue sections which will be microdissected. Only a few tissue sections designated to act as guide maps to identify the MCC zones were stained.

Following immersion of the slide in absolute ethanol, the sections were incubated in the second 100% ethanol jar to for additional two minutes and then dehydrated in xylene and air-dried as instructed and subjected immediately to LCM procedure. Furthermore, only one slide was processed at a time to minimize RNA degradation.

### 2.4. Preparation for LCM Procedure

**Figure 4:**
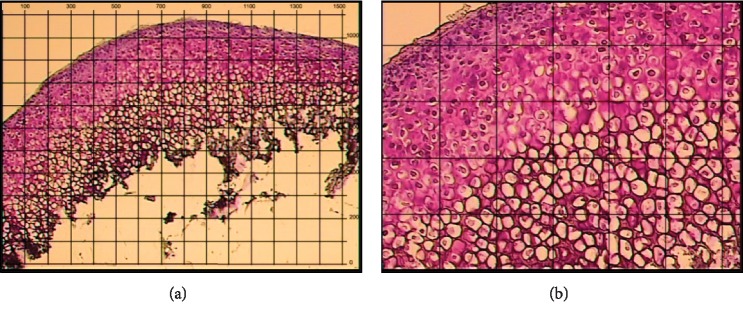
Map images of stained MCC cryosections with 100 *μ*m grid overlay. (a) Under 4x objective. (b) Under 10x objective using HistoGene Staining Solution.

The incubation block (Applied Biosystems, CA, USA) was placed inside dry heat bath with temperature set at 42°C. Meanwhile, all working surfaces, including those of the PixCell II system, and all tools were wiped and cleaned according to the RNA-free conditions (see Supplementary Materials [Supplementary-material supplementary-material-1]). To flatten the sections and to remove dust and loose tissue, PrepStrips (Applied Biosystems, CA, USA) were applied onto the section surface according to the manufacturer's instructions. In addition, the stained sections were used to identify the MCC zones through the LCM device and create images to provide guide maps when microdissecting the unstained sections if needed. At this stage, it is recommended to use the 100 *μ*m grid to roughly measure the size of cells/zones of interest (Figure 4).

### 2.5. Empirical Setting of LCM Parameters and Performing Laser Microdissection

**Figure 5:**
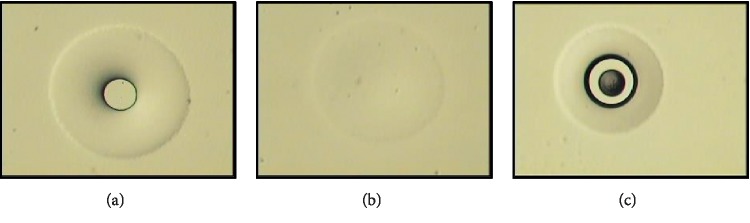
Empirical setting of LCM parameters. (a) A properly melted polymer spot has a dark outer ring and a clear center. (b) Underexposed polymer spot has a fuzzy appearance and lacks the dark ring. (c) Laser overexposure may lead to polymer burning.

**Figure 6:**
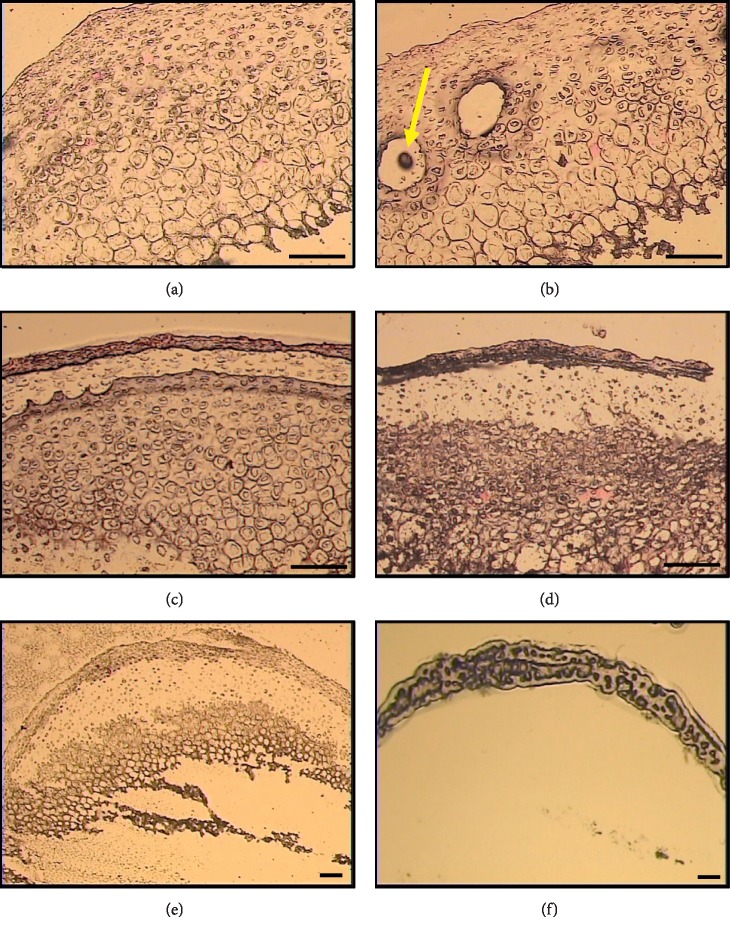
Adjustment of LCM parameters allows capturing of different sizes/areas of tissue. (a) MCC tissue section is prepared for LCM. (b) The section after LCM, where clusters of cells removed. LCM power and duration were adjusted to avoid burning (arrow) of the cap film. (c) Proper wetting permits the film to contact the selected area of the tissue section. (d) The section after LCM demonstrating capturing of a strip (continuous line) of tissue and (e) capturing of a larger zone. (f) After LCM completion, the LCM cap is examined to confirm successful microdissection of the cells of interest (scale bar = 100 *μ*m).

**Figure 7:**
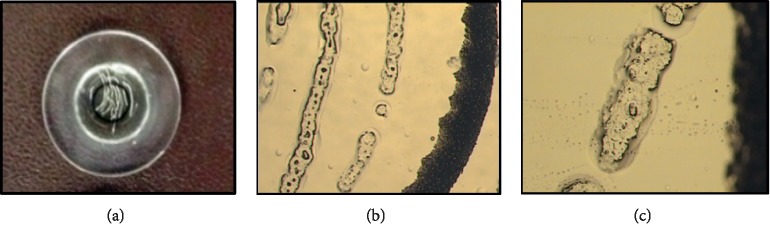
Inspection of the cap surface upon LCM completion to verify the successfully performed procedure. Microdissected tissue can be seen with the naked eye (a) and/or under the LCM microscope (b and c).

Under 10x objective, the LCM cap (Arcturus CapSureHS LCM Caps, Applied Biosystems, CA, USA) was placed on the slide outside the section area, and the laser spot size was set to 7.5 *μ*m, followed by focusing the IR laser as instructed by the manufacturer (Figures 5, 6(a), and 6(b)). Settings of the laser parameters greatly vary according to the tissue type and size of the area to be dissected. The suggested settings for the FZ are 70–90 mW power and 2-3 ms duration with 7.5 *μ*m spot size. For the PZ, MZ, and HZ, we suggest 45–65 mW and 10–15 ms, with 15 *μ*m spot size. Large areas were also dissected from the FCC using the following settings: 40–50 mW and 10–20 ms, with 30 *μ*m spot size. To perform microdissection, the guide map was reviewed, the section to be dissected was placed on the LCM stage, and the laser was continually fired until the area of interest was entirely wetted by the cap polymer (Figures 6(c) and 6(d)). We procured as many zone-specific cells/areas as possible by dissecting six sections (mounted on one slide) per cap. Documenting LCM process by taking images for the section before and after LCM (Figures 6(e) and 6(f)), as well as the cap with the microdissected tissue is recommended (Figure 7). The completeness of the capture was assessed by inspecting both the cap and the section dissected. To eliminate debris or nonspecific tissue adhering to the cap, the cap was gently pressed onto a post-it note. We dissected a second nonadjacent zone from the same slide (for instance, FZ and M or PZ and HZ) only if the sections were large enough.

### 2.6. RNA Extraction and Pooling of Cell Lysates

RNA was immediately extracted after LCM completion by transferring the cap to a 0.5 ml tube containing 50 *μ*L of lysis buffer (Arcturus® PicoPure RNA Isolation Kit, Applied Biosystems, CA, USA) as recommended by the manufacturer in the Macro Cap protocol. The cell extract was then stored at −80°C in a freezer. To achieve sufficient yield of RNA, extracts from homogeneous cell populations captured on four caps were combined onto a single RNA purification column, except for FZ samples where 8 caps were pooled together to prepare one sample to compensate for the smaller area of this zone.

### 2.7. RNA Isolation

Manufacturer's instructions were followed but with some modifications.

- Adjustment of the 70% ethanol volume to compensate for the total volume of the pooled sample (200 *μ*l)
- On-column DNase treatment (RNase-Free DNase Set, Qiagen, Germany) was done regularly for all samples as the elimination of genomic DNA is critical for accurate downstream applications
- Additional column centrifugation for 1 minute to ensure removal of the wash buffer before elution step

### 2.8. Evaluation of RNA Integrity

For evaluation of RNA integrity, we used RNA samples as template to synthesize the first-strand cDNA and then perform qRT-PCR to determine 3′/m ratio of GAPDH and *β*-Actin. Firstly, RNA samples were used as a template to synthesize first-strand cDNA according to the manufacturer's instructions using Superscript III Reverse Transcriptase (Invitrogen, CA, USA) and oligo (dT)_12–18_ (Invitrogen, CA, USA). 10 *μ*L of LCM-RNA was used per 20 *μ*L cDNA synthesis reaction using Veriti 96-well thermal cycler (Applied Biosystems, CA, USA). To assess RNA quality, 3′/m ratio of GAPDH and *β*-Actin was determined. For this, two primers sets, one specific for the 3′ end and the other one for the middle (m) region of each housekeeping gene, were designed using Primer Express 3.0 (Table 1).

Standard curves were generated for each primer pair with serial dilutions of cDNA synthesized using RNA isolated from a mixture of MCC and FCC specimens. These specimens were dissected and separated from the subchondral bone with a scalpel, placed and wrapped with tinfoil, dipped into liquid nitrogen to solidify, and then ground to a fine tissue powder. Total RNA was isolated and purified in accordance with the manufacturer's instructions (RNeasy Fibrous Tissue Mini Kit, Qiagen, Germany). The amount of isolated total RNA was quantified using the Nanodrop (OD 260/280 was 2.05, and OD 260/230 was 2.11), and then aliquots of 5 *μ*l were prepared so as not to exceed the maximum (5 *μ*g) recommended total RNA quantity for the subsequent reverse transcription procedure. The isolated genetic material was used as a template to synthesize the first-strand cDNA using Superscript III Reverse Transcriptase (Invitrogen, CA, USA) and oligo (dT)_12–18_ (Invitrogen, CA, USA) according to the manufacturer instructions. The qRT-PCR was performed using a Step One Plus RT-PCR system (Applied Biosystems, CA, USA) and Power SYBR® Green PCR master mix (Applied Biosystems, Warrington, UK). Relative quantities for the four tested regions were determined utilizing the corresponding standard curves generated in the same experiment, and then the 3′/m ratio was calculated for each sample.

## 3. Results

### 3.1. Histological Comparison

**Figure 8:**
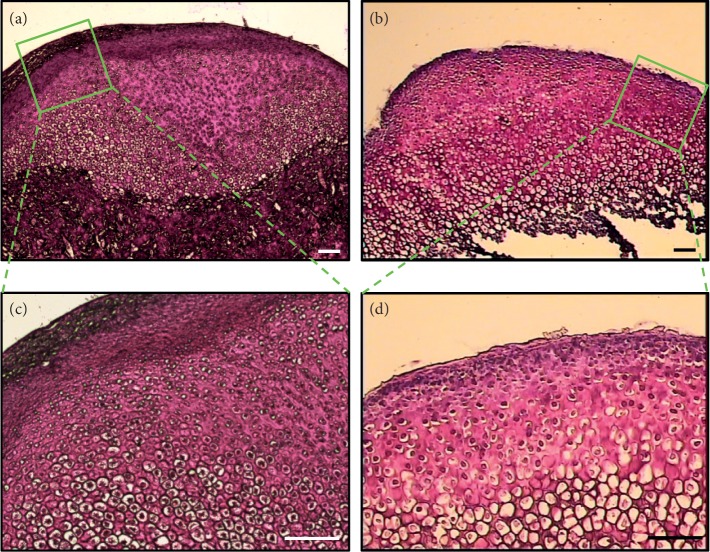
Comparison of images taken for stained FFPE (a and c) and frozen (b and d) tissue sections demonstrated equally clear morphological details that permit identification of the different cell populations of MCC (scale bar = 100 *μ*m).

With the two methods applied, namely, formalin fixation and cryopreservation using precooled isopentane, the stained tissue sections showed no discernible histological differences in the quality of images, and both methods resulted in a staining pattern good enough to reliably identify the different zones (Figure 8). This finding, together with the superior quality of RNA extracted, justifies the adoption of the cryopreservation method in this protocol.

### 3.2. Cryosectioning

Under the settings recommended in this protocol, smooth tissue sections with minimal tearing and folding were obtained. This outcome facilitated both the histological examination and LCM application. Right and left MCC specimens of the same animal were mounted together in one cryomold; therefore, one cryosection contained two sections. This enabled us to mount 6 tissue sections (3 cryosections), 7*μ*m thick each per slide within a time period shorter than that needed for 6 cryosections, resulting in a greater chance to avoid RNA degradation. In addition, to avoid subjecting the OCT-embedded specimens to multiple freeze-thaw cycles, the whole OCT block was cryosectioned at one time, producing >400 MCC sections mounted on 70 slides (6 sections/slide). Only one tissue section is mounted on every tenth slide, and then stained to provide a guide map during LCM procedures. The first ten and the last ten slides were not used for LCM procedure because the sections were small in size and did not allow reliable identification of the MCC zones. The middle 30–40 slides contained relatively large tissue sections, allowing homogenous isolation of cells from two zones separately using two LCM caps.

### 3.3. Staining

**Figure 9:**
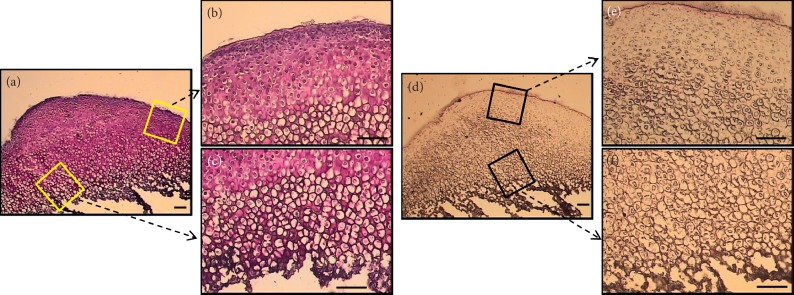
Images of stained (a, b, and c) and unstained (d, e, and f) frozen sections of MCC tissue taken directly on the LCM instrument without coverslip; both types of sections allowed clear distinction between MCC zones (scale bar = 100 *μ*m).

In line with the RNase-free recommendations, ProtectRNA was added to the dehydrating solutions except xylene. This causes the solutions to be discolored to orange/red, resulting in “faint staining” to the sections. When examined under the microscope, these unstained sections revealed morphological details just clear enough to allow distinction between the four MCC zones (Figure 9). Therefore, we skipped tissue sections staining and the following washing steps in our protocol except for the guide-map sections.

### 3.4. Verification of LCM Efficiency

**Figure 10:**
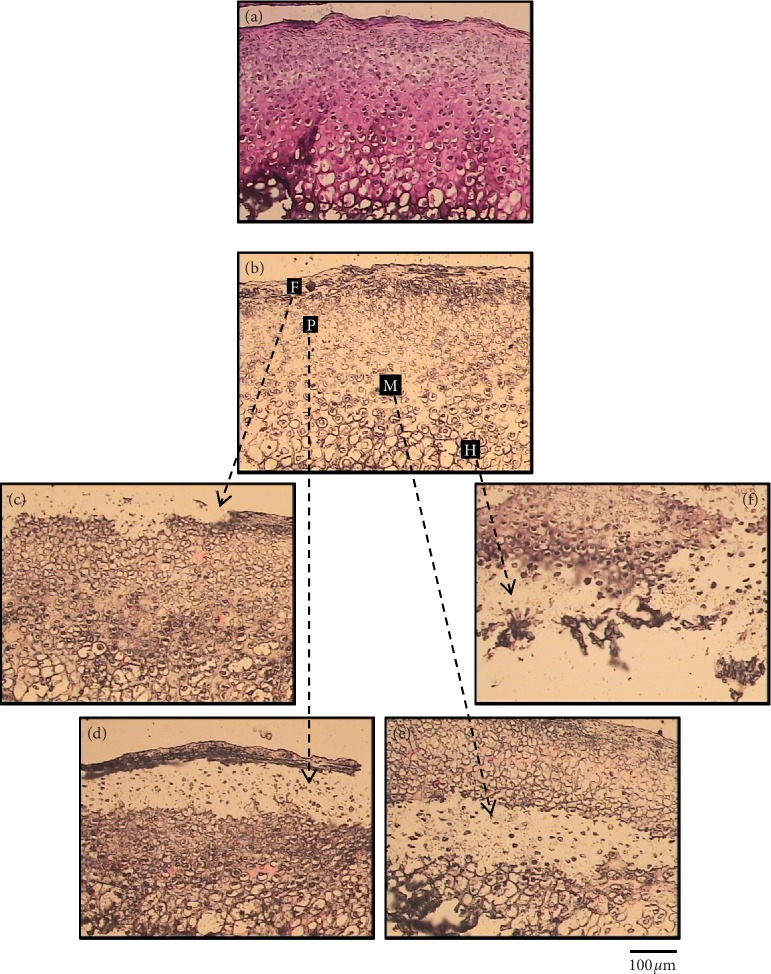
Zonal isolation of homogenous cell populations from the MCC tissue. (a) Index-matched image is used to guide the LCM process. (b) Unstained, dehydrated, frozen MCC tissue section before LCM demonstrating the four zones (FZ, MZ, PZ, and HZ). (c–f) MCC sections after LCM where cells were individually isolated from their respective zones.

Selection of laser spot size and the setting values depends largely on the size of cells/zones to be dissected, and thus rough measurements for the MCC zones were obtained. The widest diameter for the MCC of a 5-week-old rat was approximately 3 mm (Figure 1(c)), while the depth of the FZ, PZ, MZ, and HZ was roughly 10–20, 50–100, 50–200, and 100–300 *μ*m, respectively (Figure 4). Likewise, the FCC specimen was around 3-4 mm in diameter (Figure 1(e)), but with relatively greater depth of about 500–800 *μ*m. Using the empirically set LCM parameters, we efficiently performed LCM for different areas starting from clusters of cells (Figure 6(b)), to very narrow tissue strips (a few *μ*m-wide continuous lines) such as FZ (Figure 6(d)), to slightly wider strips (up to a few hundreds of *μ*m) such as PZ, MZ, and HZ (Figure 6(e)). Besides knowing the size of the selected area, correct adjustment of laser power and duration for each section is of paramount importance for efficient LCM. We found that the laser parameters used to microdissect the PZ could be also applied to MZ and HZ, whereas FZ could be successfully captured after some adjustment of the laser settings as described in the methodology section (Figure 10).

### 3.5. Assessment of RNA Integrity

**Figure 11:**
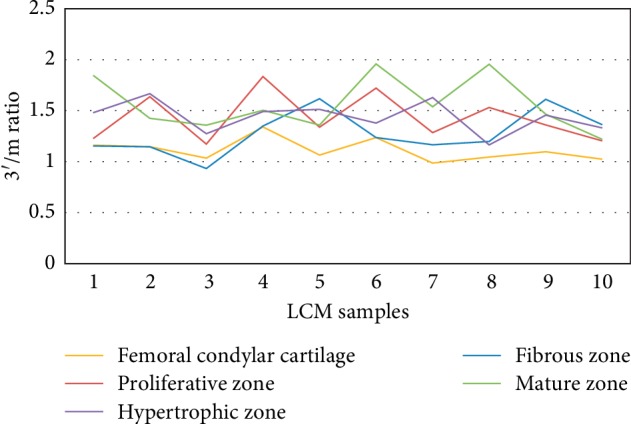
3′/m ratio determined by RT-qPCR as a measure of the quality of LCM-RNA samples for GAPDH.

**Figure 12:**
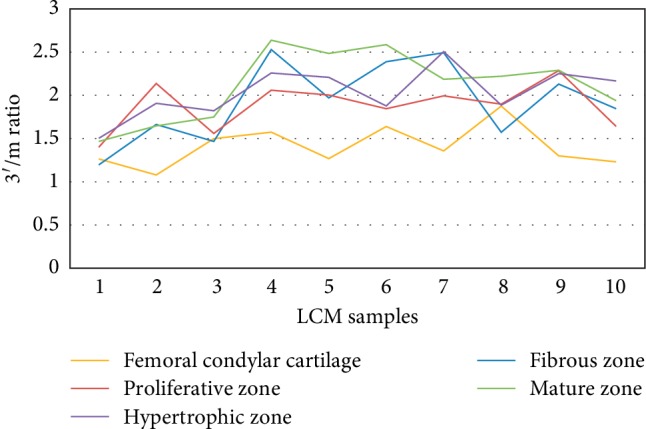
3′/m ratio determined by RT-qPCR as a measure of the quality of LCM-RNA samples for *β*-Actin.

The analysis of RNA quality was based on qRT-PCR with GAPDH and *β*-Actin primers for 3′ end and the middle (m) regions. The results showed that the ranges of 3′/m GAPDH for the C, FZ, PZ, MZ, and HZ groups were 0.99–1.34, 0.93–1.36, 1.17–1.83, 1.22–1.96, and 1.16–1.67, respectively (Figure 11). On the other hand, the 3′/m *β*-Actin ranged as follows: 1.08–1.88, 1.20–2.53, 1.40–2.29, 1.47–2.64, and 1.50–2.26 for the same order of groups, respectively (Figure 12).  Table 2 shows the 3′/m mean ± standard deviation of the two housekeeping genes for the five groups. The average values of the 3′/m of all MCC samples (40 LCM samples) were 1.43 and 1.99 for GAPDH and *β*-Actin, respectively.

## 4. Discussion

LCM technique has been applied to a variety of tissues using different methods to prepare a wide range of biological samples. Contrary to DNA, RNA is more sensitive to specimen handling and preparation procedure and requires strictly followed RNase-free techniques [7]. Several protocols have been suggested to optimize the procedure for LCM-RNA recovery and to cope with technical challenges [8, 15–25]. One of the challenges is that LCM process is lengthy and performed at room temperature. It may also require tissue staining, which exposes RNA to chemical components and aqueous solutions. Moreover, microdissecting multiple zones from the same tissue section and pooling procedures make the challenge greater [20]. In addition, because of the high cost, the LCM instrument is usually a core-facility service separately located from the histology facility. Logistic issues such as limited access time to the LCM instrument and transporting samples between facilities may further complicate the process [7]. Therefore, when conducting LCM experiments for RNA recovery, there are two requirements: the first is to obtain acceptable tissue section morphology that allows histological identification of the desirable cell/tissue type, and the second is to preserve the integrity and biological accessibility to RNA. Each step in the LCM protocol could have a serious impact on RNA quality, and thus optimization of some crucial factors is highly important.

### 4.1. Factors Enhancing Tissue Section Visualization and RNA Integrity

#### 4.1.1. Specimen Freezing

Formalin is the standard fixative in histology laboratories. However, it can create extensive cross-links with the proteins and nucleic acids, and consequently the recovered macromolecules will be highly fragmented [16, 26]. On the other hand, specimen freezing maximizes both the RNA quality and quantity [8, 27, 28], but it may disrupt tissue histology [26]. When a specimen is directly immersed in liquid nitrogen, ice crystals might be formed within the tissue destroying its histological details, whereas freezing at a slower rate (by using precooled isopentane in dry ice) can maintain RNA integrity and provide histological details close to that achieved with FFPE sections [13, 29]. Our histological findings confirmed this point where both FFPE and unfixed frozen MCC sections revealed equally clear details that allow reliable distinction of the histological zones (Figure 8). Since this freezing method fulfills the requirements of preserving both tissue morphology and RNA integrity, it was chosen for tissue preparation in this study. This method involves the following steps: place both right and left specimens in one cryomold; freeze the prepared cryomold into the cooled isopentane; set the cryostat temperature to −24°C to −30°C.

#### 4.1.2. Elimination of Staining Step

The ideal stain should be compatible with both the macromolecules retrieved and the subsequent molecular assay, and it should also provide good morphology [26]. However, the stain chemicals may affect the integrity of the macromolecules, and the aqueous component of the stain may activate RNases [22]. Moreover, our preliminary work demonstrated increased chances of section detachment associated with the staining procedure. Using a stain with less intensity [13, 27] for shorter incubation time [7, 13, 16, 20] and addition of RNase inhibitor to the staining solutions [16, 17, 22, 24] allows RNA recovery of high quality. Another choice is to use unstained dehydrated sections for LCM if the histological features are sufficient to identify the zones of interest [30]. Nonetheless, when the section is completely dry and uncovered, it appears as a gray-scale image because the color and details are not refracted [16, 22, 31]. This visual limitation is further complicated by the fact that coverslips and immersion oils are not compatible with the LCM device because the cap should have direct physical access to the tissue to be dissected [16]. In our study, visualization of tissue details was not limited probably due to the normal histological features of the MCC tissue which allowed relatively easy identification of the zonal structure, the use of ProtectRNA which resulted in “faint staining” effect (Figure 9), and/or using the digitally saved images of matching stained sections as a guide to locate the areas of interest.

### 4.2. Factors Enhancing LCM Efficiency and RNA Integrity

#### 4.2.1. Optimizing Cryosectioning Parameters

Tissue section thickness, type of slides used, number of sections mounted per slide, and cryostat temperature can play a role and may have an impact on the LCM efficiency and quality of the extracted RNA. Many users recommended 5–8 *μ*m range for section thickness; below 5 *μ*m may be less than cell thickness interfering with the correct identification of cells to be captured and necessitating recruitment of more cells for the subsequent assay, while above 8 *μ*m thickness may lead to incomplete microdissection and compromised tissue lifting [15–17, 26, 31].

Tissue sections can be mounted on plain, superfrosted, charged/coated, or membrane microscopic slides depending on specimen type and microdissection method [32]. Charged slides are usually not used for LCM (IR laser) because of the stronger attraction between this type of slides and tissue sections which can result in LCM failure [16, 17]. However, tissues with open architecture such as lungs and bones are usually mounted on coated slides to avoid nonspecific adherence of loose tissue to the LCM cap [16]. In addition, our preliminary work showed loss of 1-2 sections/slide during staining and dehydration procedures. Therefore, we used charged slides to reduce sections loss and to minimize the nonspecific adherence of condylar bone.

Previous studies reported a range from −17°C to −20°C for the cryostat temperature [17, 18, 20], while ours is ranging from −24°C to −30°C. This range is in agreement with Fukui et al. who used −20°C to −30°C temperature to prepare FCC cryosections with the healthy specimens requiring lower temperature (toward −30°C) as compared with diseased ones [30]. Additionally, this range reduces the temperature gradient between the deep freezer and the cryostat chamber, thus maintaining RNA integrity.

The concept of preparing one slide with multiple sections is introduced to compensate for section loss problem during staining and dehydration procedures [17, 30] and to allow maximizing RNA quantity by dissecting as much as possible of desirable cells from several small tissue sections [33]. To take the advantage of this concept yet minimizing RNA degradation, specimen cutting and section mounting should be performed as rapidly as possible. Therefore, we suggested embedding two (right and left) MCCs in one cryomold, so that only three cryocuts are needed to mount six tissue sections onto one slide.

#### 4.2.2. Efficient Dehydration

Tissue dehydration is important not only for LCM success via minimizing the adhesive forces between the slide surface and the section [16] but also for RNA stabilization by inhibiting endogenous RNases of the tissue [7, 8]. Accordingly three measures, which have been described in previous LCM protocols [8, 17, 31], were considered in this study: adding RNase inhibitor (ProtectRNA) to all dehydration solutions except xylene (because of insolubility), adding molecular sieves for both xylene and 100% ethanol during the final dehydration steps, and extending the incubation time to 2 minutes in the second 100% ethanol jar.

#### 4.2.3. Optimizing LCM Parameters

When firing laser, the polymer film of the LCM cap melts focally. In case of proper melting, the film appears under the LCM microscope as a clear circular center surrounded by a dark ring (Figure 5(a)) [15, 16, 31]. A fuzzy ring (Figure 5(b)) indicates a poorly wetted spot which could be attributed to an improperly seated cap, unfocused laser, or inadequate power and/or duration of the laser [15, 31]. The wide range of laser power and duration values [8, 16, 26, 30] emphasizes the need to empirically set the LCM parameters for each experiment. For efficient zonal LCM of MCC tissue (Figure 10), it is recommended to know the area of the zone to be dissected and secondly to adjust laser power and duration accordingly for each section. Yet, LCM failure may be encountered if cryosections were allowed to dry at room temperature, resulting in increased adherence forces to the slide [7, 26]. LCM failure could also be attributed to incompletely dehydrated sections or insufficient laser settings [7, 26].

#### 4.2.4. LCM Time Limit

LCM samples are microdissected at room temperature, thus it should be completed as rapid as possible to conserve the high quality of RNA. Some authors recommended 30 minutes period of time for staining, dehydration, and microdissection, followed by immediate extraction of RNA [16, 24, 30]. However, when dissecting two zones from several tissue sections, time and temperature may have serious impact on the quality of the extracted RNA [18]. A time limit of a maximum of 3 hours between tissue staining and cell lysis has been reported by Stemmer et al. In our experiment, it lasted less than 30 minutes to dehydrate and microdissect one zone from 6 MCC sections mounted on one slide, probably because the MCC sections are small in area and the zone selected for LCM is even much smaller (∼50–500 *μ*m^2^) (Figure 4). However, a slightly longer time was needed to harvest two cell groups from the same slide. The overall range of time was 30 ± 15 minutes, a range that has been previously reported [20, 24]. Familiarity with the protocol steps and troubleshooting management is crucial to accomplish the approach recommended in our protocol starting with removing the cryosections from the deep freezer till the immersion of the LCM cap with captured tissue in the lysis buffer for RNA extraction.

### 4.3. Factors Enhancing LCM Specificity

PixCell II LCM instrument is compatible with two types of LCM caps: macro caps and HS caps. The former type has four times the area of an HS cap available for microdissection, while the latter has a 12 *μ*m rail to prevent the polymer film from touching the dehydrated section, therefore limiting any possible contamination from nonselected adjacent cells [16]. As recommended by Pietersen et al. [17], we used the HS cap to take the advantage of the ridge, but as a macro cap. In addition, the homogeneity of selected cells was enriched by using the Prepstrips to flatten the tissue section and eliminate loose tissue fragments [8, 23]. The third measure we took to enhance LCM specificity was to clean the cap after completion of the microdissection process to remove any debris or nonspecific tissue adhering to the cap.

### 4.4. Factors Enhancing RNA Yield

Typically, LCM samples are so small that minute amount of RNA is recovered, and great variability in the yields from different types of cells has been reported [32, 34] with a range of 1–20 pg/cell (Table 3). To achieve the recommended amounts of LCM-RNA required for subsequent downstream analysis, samples were pooled on two levels. Firstly, six cryosections were mounted on one slide, and then LCM is carried out to microdissect the areas of interest from the six mounted sections using one cap [18, 20]. However, some research groups are concerned with keeping the cap at room temperature, while microdissection of several sections is completed [22, 30]. For MCC tissue, the overall area of the whole section is relatively small, and the area of the zones to be dissected is even much smaller as mentioned above. Further enhancement of the LCM-RNA yield was achieved by a second level of pooling, whereby several lysates of the same cell phenotype were added to one RNA purification column [20, 24]. Sample pooling protocol in this study is similar to that of Wang et al. [20], where 4 cell lysates retrieved from the same cell population were pooled and added to one extraction column. Moreover, the cells of each lysate were collected by performing LCM for 4–6 tissue sections mounted on one slide.

The smaller the number of cells in LCM samples, the higher the RNA loss during isolation [32, 34]. To limit this undesirable loss, we considered two measures: LCM procedure was immediately followed by RNA extraction to stabilize RNA, in addition, one of the column-based special kits for isolation of RNA in picograms was employed.

### 4.5. Quality Control of the LCM-RNA Samples

The reliability of the subsequent molecular analysis is largely affected by the quality of RNA extracted [27, 35]. For minute LCM-RNA samples, conventional assessment methods of RNA quality have a tendency to produce inconsistent and imprecise results probably because the quantity of RNA is below the sensitivity range of the technology used [35–37]. Therefore, 3′/m ratio for housekeeping genes has been suggested as a metric measure to evaluate qualitative performance of small RNA samples [24, 38]. Kube et al. [24] developed qPCR assay to measure the 3′/m ratio. Since the qPCR method is more sensitive and requires low sample concentrations, the results were reproducible. Moreover, the 3′/m ratio allows direct functional assessment of mRNA rather than calculating 28S : 18S of rRNA as a surrogate to mRNA [24]. The 3′/m ratio for a gene measures the abundance of that gene at the 3′ end to the abundance at the midregion. In the presence of nondegraded full-length transcripts, the two quantities at the 3′ and midregions are almost equal, resulting in a ratio close to 1, whereas shortened/degraded transcripts show less PCR amplification at (m) region and hence higher 3′/m ratio [24].

Our results revealed 3′/m averages of 1.1–1.6 and 1.4–2.1 for GAPDH and *β*-Actin, respectively (Table 2). There are no fixed cut-off values for the 3′/m ratio, as different studies could tolerate different ratios; however, some reported values may work as a guide. For LCM samples, Kube et al. have chosen a cutoff value of ≤2 for 3′/m ratio of *β*-Actin [24]. In our study, the overall average ratios of 40 MCC samples were 1.43 and 1.99 for GAPDH and *β*-Actin, respectively; these values are in line with the reported quality control requirements. Thus, the LCM protocol we described allowed preparation of LCM-RNA samples that meet quality requirements of further molecular analyses.

## 5. Conclusions

We described a protocol for IR-LCM of four zone-specific cell populations from the MCC heterogeneous tissue. The use of our optimized protocol could allow:

- Preparation of unfixed and undecalcified MCC cryosections with histomorphological features comparable with FFPE sections.
- Efficient isolation of homogenous cell-specific populations from each of the four MCC zones, namely, fibrous, proliferative, mature chondrocyte, and hypertrophic chondrocyte zones.
- Obtaining RNA samples with high quality, thus allowing accurate subsequent downstream analysis. Generating accurate cellular, molecular, and genetic data facilitates developing a specific molecular signature and fingerprints for cell population or a specific pathological lesion or condition. This information, in turn, provides invaluable insights for MCC tissue-engineering field and regenerative cell-based therapy.

## Figures and Tables

**Table 1:** Sequence of primer sets for housekeeping genes used for qRT-PCR to evaluate LCM-RNA integrity.

Gene symbol	Forward primer sequence (5′-3′)	Reverse primer sequence (5′-3′)
GAPDH (3′ end)	ATGTATCCGTTGTGGATCTGACAT	AGCCCAGGATGCCCTTTAGT
GAPDH (midregion)	CCTCAAGATTGTCAGCAATGCA	GGCATGGACTGTGGTCATGA
*β*-Actin (3′ end)	GCTCCTCCTGAGCGCAAGT	CATCGTACTCCTGCTTGCTGAT
*β*-Actin (midregion)	GGCCAACCGTGAAAAGATGA	ACCAGAGGCATACAGGGACAA

**Table 2:** Average values of 3′/m GAPDH and *β*-Actin ratio for LCM-RNA samples retrieved from groups C (the control), FZ, PZ, MZ, and HZ.

Group	3′/m GAPDH (mean ± SD)	3′/m *β*-Actin (mean ± SD)
C (femoral condylar cartilage)	1.11 ± 0.11	1.41 ± 0.24
FZ (fibrous zone of MCC)	1.28 ± 0.21	1.93 ± 0.46
PZ (proliferative zone of MCC)	1.43 ± 0.23	1.88 ± 0.28
MZ (mature zone of MCC)	1.56 ± 0.26	2.12 ± 0.41
HZ (hypertrophic zone of MCC)	1.44 ± 0.15	2.04 ± 0.29

**Table 3:** Variation in cellular RNA yields (in picograms) from LCM samples.

Reference	Amount of RNA recovery/cell (pg)
Pietersen et al. [17]	1
Wang et al. [20]	2.5
Peterkova et al. [34]	4.5–11.8
Ladanyi et al. [32]	10
Espina et al. [16]	10
HistoGene™ LCM frozen section staining kit userguide/AB	20

## Data Availability

The data used to support the findings of this study are included within the article.

## Supplementary Materials

Supplementary Materials Supplementary file S1: ARRIVE guidelines checklist. Supplementary file S2: RNase-free technique.

## References

[1] Wang L., Detamore M. S. (2007). Tissue engineering the mandibular condyle. *Tissue Engineering*.

[2] Chen J., Utreja A., Kalajzic Z., Sobue T., Rowe D., Wadhwa S. (2012). Isolation and characterization of murine mandibular condylar cartilage cell populations. *Cells Tissues Organs*.

[3] Tanaka E., Detamore M. S., Mercuri L. G. (2008). Degenerative disorders of the temporomandibular joint: etiology, diagnosis, and treatment. *Journal of Dental Research*.

[4] Dibbets J. M. H., Carlson D. S. (1995). Implications of temporomandibulardisorders for facial growth and orthodontic treatment. *Seminars in Orthodontics*.

[5] Murakami T., Fukunaga T., Takeshita N. (2010). Expression of Ten-m/Odz3 in the fibrous layer of mandibular condylar cartilage during postnatal growth in mice. *Journal of Anatomy*.

[6] Kuroda S., Tanimoto K., Izawa T., Fujihara S., Koolstra J. H., Tanaka E. (2009). Biomechanical and biochemical characteristics of the mandibular condylar cartilage. *Osteoarthritis and Cartilage*.

[7] Fend F., Raffeld M. (2000). Laser capture microdissection in pathology. *Journal of Clinical Pathology*.

[8] Clement-Ziza M., Munnich A., Lyonnet S., Jaubert F., Besmond C. (2008). Stabilization of RNA during laser capture microdissection by performing experiments under argon atmosphere or using ethanol as a solvent in staining solutions. *RNA*.

[9] Darling E. M., Hu J. C. Y., Athanasiou K. A. (2004). Zonal and topographical differences in articular cartilage gene expression. *Journal of Orthopaedic Research*.

[10] Kim T.-K., Sharma B., Williams C. G. (2003). Experimental model for cartilage tissue engineering to regenerate the zonal organization of articular cartilage. *Osteoarthritis and Cartilage*.

[11] Shieh A. C., Athanasiou K. A. (2006). Biomechanics of single zonal chondrocytes. *Journal of Biomechanics*.

[12] Landesberg R., Proctor R. L., Rosier R. N., Puzas J. E. (1995). The mandibular condylar growth center: separation and characterization of the cellular elements. *Calcified Tissue International*.

[13] Pinzani P., Orlando C., Pazzagli M. (2006). Laser-assisted microdissection for real-time PCR sample preparation. *Molecular Aspects of Medicine*.

[14] Hinton R., Serrano M., So S. (2009). Differential gene expression in the perichondrium and cartilage of the neonatal mouse temporomandibular joint. *Orthodontics & Craniofacial Research*.

[15] Espina V., Milia J., Wu G., Cowherd S., Liotta L. A., Taatjes D. J., Mossman B. T. (2006). Laser capture microdissection. *Methods Molecular Biologyd/Cell Imaging Techniques: Methods and Protocols*.

[16] Espina V., Wulfkuhle J. D., Calvert V. S. (2006). Laser-capture microdissection. *Nature Protocols*.

[17] Pietersen C. Y., Lim M. P., Woo T. U. (2009). Obtaining high quality RNA from single cell populations in human postmortem brain tissue. *Journal of Visualized Experiments*.

[18] Stemmer K., Ellinger-Ziegelbauer H., Lotz K., Ahr H.-J., Dietrich D. R. (2006). Establishment of a protocol for the gene expression analysis of laser microdissected rat kidney samples with affymetrix genechips. *Toxicology and Applied Pharmacology*.

[19] Tachikawa T., Irie T. (2004). A new molecular biology approach in morphology: basic method and application of laser microdissection. *Medical Electron Microscopy*.

[20] Wang W.-Z., Oeschger F. M., Lee S., Molnár Z. (2009). High quality RNA from multiple brain regions simultaneously acquired by laser capture microdissection. *BMC Molecular Biology*.

[21] Mikulowska-Mennis A., Taylor T. B., Vishnu P. (2002). High-quality RNA from cells isolated by laser capture microdissection. *Biotechniques*.

[22] Wang H., Owens J. D., Shih J. H., Li M.-C., Bonner R. F., Mushinski J. F. (2006). Histological staining methods preparatory to laser capture microdissection significantly affect the integrity of the cellular RNA. *BMC Genomics*.

[23] Barcala M., Fenoll C., Escobar C., Gassmann W., Jin H. (2012). Laser microdissection of cells and isolation of high-quality RNA after cryosectioning. *Methods Molecular Biology/RNA Abundance Analysis: Methods and Protocols*.

[24] Kube D. M., Savci-Heijink C. D., Lamblin A.-F. (2007). Optimization of laser capture microdissection and RNA amplification for gene expression profiling of prostate cancer. *BMC Molecular Biology*.

[25] Upson J. J., Stoyanova R., Cooper H. S. (2004). Optimized procedures for microarray analysis of histological specimens processed by laser capture microdissection. *Journal of Cellular Physiology*.

[26] Decarlo K., Emley A., Dadzie O. E., Mahalingam M., Murray G. I. (2011). Laser capture microdissection: methods and applications. *Methods Molecular Biology/Laser Capture Microdissection: Methods and Protocols*.

[27] Goldsworthy S. M., Stockton P. S., Trempus C. S., Foley J. F., Maronpot R. R. (1999). Effects of fixation on RNA extraction and amplification from laser capture microdissected tissue. *Molecular Carcinogenesis*.

[28] Hiller T., Snell L., Watson P. H. (1996). Microdissection RT-PCR analysis of gene expression in pathologically defined frozen tissue sections. *Biotechniques*.

[29] Huang L. E., Luzzi V., Ehrig T., Holtschlag V., Watson M. A. (2002). Optimized tissue processing and staining for laser capture microdissection and nucleic acid retrieval. *Methods in Enzymology*.

[30] Fukui N., Ikeda Y., Tanaka N., Murray G. I. (2011). The use of laser capture microdissection on adult human articular cartilage for gene expression analysis. *Methods Molecular Biology/Laser Capture Microdissection: Methods and Protocols*.

[31] Gallagher R. I., Blakely S. R., Liotta L. A., Espina V., Espina V., Liotta L. A. (2012). Laser capture microdissection: arcturus (XT) infrared capture and UV cutting methods. *Methods Molecular Biology/Molecular Profiling: Methods and Protocols*.

[32] Ladanyi A., Sipos F., Szoke D., Galamb O., Molnar B., Tulassay Z. (2006). Laser microdissection in translational and clinical research. *Cytometry Part A*.

[33] Field L. A., Deyarmin B., Shriver C. D., Ellsworth D. L., Ellsworth R. E., Murray G. I. (2011). Laser microdissection for gene expression profiling. *Methods Molecular Biology/Laser Capture Microdissection: Methods and Protocols*.

[34] Peterková M., Koutná I., Tesarová L. (2009). Microarray analysis using a limited amount of cells. *Folia Biologica*.

[35] Copois V., Bibeau F., Bascoul-Mollevi C. (2007). Impact of RNA degradation on gene expression profiles: assessment of different methods to reliably determine RNA quality. *Journal of Biotechnology*.

[36] Clément-Ziza M., Gentien D., Lyonnet S., Thiery J.-P., Besmond C., Decraene C. (2009). Evaluation of methods for amplification of picogram amounts of total RNA for whole genome expression profiling. *BMC Genomics*.

[37] Imbeaud S., Graudens E., Boulanger V. (2005). Towards standardization of RNA quality assessment using user-independent classifiers of microcapillary electrophoresis traces. *Nucleic Acids Research*.

[38] Wilson C. L., Pepper S. D., Hey Y., Miller C. J. (2004). Amplification protocols introduce systematic but reproducible errors into gene expression studies. *Biotechniques*.

