# Supplementary material for: Optimizing Laser Capture Microdissection Protocol for Isolating Zone-Specific Cell Populations from Mandibular Condylar Cartilage
Source: Int J Dent. 2019 Nov 21;2019:5427326. doi: 10.1155/2019/5427326 (PMC6914897; doi:10.1155/2019/5427326)
Supplement: Supplementary Materials — Supplementary file S1: ARRIVE guidelines checklist. Supplementary file S2: RNase-free technique. [file 5427326.f1.docx]

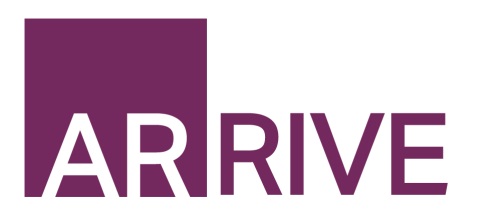


***S1 SUPPLEMENTARY FILE***

The ARRIVE Guidelines Checklist

Animal Research: Reporting In Vivo Experiments

Carol Kilkenny^1^, William J Browne^2^, Innes C Cuthill^3^, Michael Emerson^4^ and Douglas G Altman^5^

*^1^The National Centre for the Replacement, Refinement and Reduction of Animals in Research, London, UK, ^2^School of Veterinary Science, University of Bristol, Bristol, UK, ^3^School of Biological Sciences, University of Bristol, Bristol, UK, ^4^National Heart and Lung Institute, Imperial College London, UK, ^5^Centre for Statistics in Medicine, University of Oxford, Oxford, UK.*

|  | | ITEM | RECOMMENDATION | Section/ Paragraph |
| --- | --- | --- | --- | --- |
| 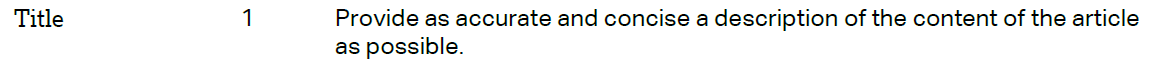 | | | **Optimizing laser-capture microdissection protocol for isolating zone-specific cell populations from mandibular condylar cartilage** |  |
| 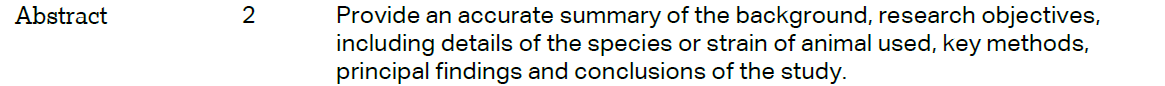 | | | Mandibular condylar cartilage (MCC) is a multi-zonal heterogeneous fibrocartilage consisting of: fibrous (FZ), proliferative (PZ), mature (MZ) and hypertrophic (HZ) zones. Gross sampling of the whole tissue may conceal some important information and compromise the validity of the molecular analysis. Laser-capture microdissection (LCM) technology allows isolating zonal (homogenous) cell populationsand consequently generating more accurate molecular and genetic data, but the challenges during tissue preparation and microdissection procedures are to obtain acceptable tissue section morphology that allows histological identification of the desirable cell type and to minimize RNA degradation. Therefore, our aim is to optimize an LCM protocol for isolating four homogenous zone-specific cell populations from their respective MCC zones while preserving the quality of RNA recovered.  MCC and FCC (femoral condylar cartilage) specimens were harvested from 5-week-old Sprague-Dawley male rats. Formalin-fixed and frozen unfixed tissue sections were prepared and compared histologically. Additional specimens were microdissected to prepare LCM samples from FCC and each MCC zone individually. Then, to evaluate LCM- RNA integrity, 3'/m ratios of Glyceraldehyde 3-phosphate dehydrogenase (GAPDH) and Beta-Actin (β-Actin) using quantitative reverse transcription-polymerase chain reaction (qRT-PCR) were calculated. Both fixed and unfixed tissue sections allowed reliable identification of MCC zones. The improved morphology of the frozen sections of our protocol has extended the range of cell types to be isolated. Under the empirically set LCM parameters, four homogeneous cell populations were efficiently isolated from their respective zones. The 3'/m ratio means of GAPDH and β-Actin ranged between 1.11-1.56 and 1.41-2.12 respectively. These values are in line with the reported quality control requirements.  The present study shows that the optimized LCM protocol could allow isolation of four homogenous zone-specific cell populations from MCC, meanwhile preserving RNA integrity to meet the high quality requirements for subsequent molecular analyses. Thereby, accurate molecular and genetic data could be generated. |  |
| INTRODUCTION | | |  |  |
| 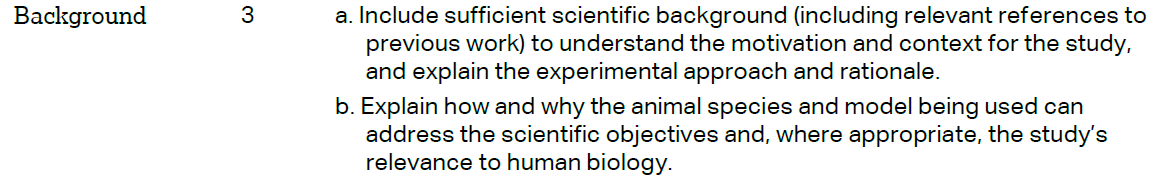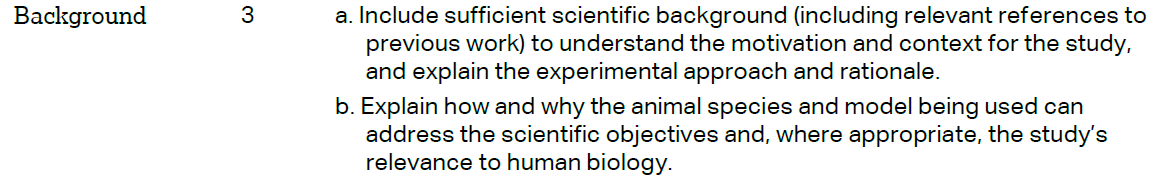 | | | While the reliability of molecular studies heavily rely on the procurement of homogenous cell populations [[7](#_ENREF_7)], native tissues are inherently heterogeneous. Traditional gross sampling would eventually result in average values of different cell phenotypes, concealing important information, and probably questioning the validity of the subsequent analysis [[8](#_ENREF_8)]. Manual microdissection [[9-11](#_ENREF_9)] may allow zonal isolation from the tissue studied, but it is time-consuming, and requires excellent manual skills. This technique is not applicable for the small-sized animals like rats and mice. In addition, isolation of several zones from MCC via this technique is not possible for its relatively smaller size, as well as its irregular zonal distribution [[7](#_ENREF_7)]. Other non-manual techniques have been attempted to isolate the MCC zones, such as countercurrent centrifugal elutriation [[12](#_ENREF_12)], and fluorescence-activated cell sorting technique [[2](#_ENREF_2)], but the former technique is not very sensitive, and the latter one requires to culture the assorted cells prior to gene expression profiling. Laser-capture microdissection (LCM) technology, on the other hand, allows precise procurement of cells of interest from a heterogeneous tissue rapidly and in a practical manner [[13](#_ENREF_13)]. Murkami *et al* (2010) succeeded to use infra-red (IR) LCM to procure two groups of cells; one from the superficial fibrous-like layer (FZ & PZ), and the other one from the deeper cartilage-like layer (MZ & HZ) of the MCC [[5](#_ENREF_5)]. Therefore, it was not possible to discern which subpopulation of cells in the same group is the source of the data obtained [[14](#_ENREF_14)].  As numerous properties are shared, Sprague-Dawley 5-week-old male rat (Rattus norvegicus) was chosen as an experimental model. We selected the age of 5 weeks not only because MCC articulation function is already present in a more mature state, but also the maximum growth spurt for rats occurs at day 31.5. |  |
| 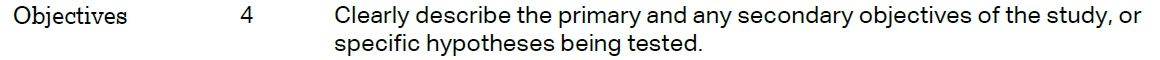 | | | The aim of this study is to optimize a protocol for isolating several homogenous cell populations from their respective MCC zones of rats using LCM technique. |  |
| METHODS | | |  |  |
| 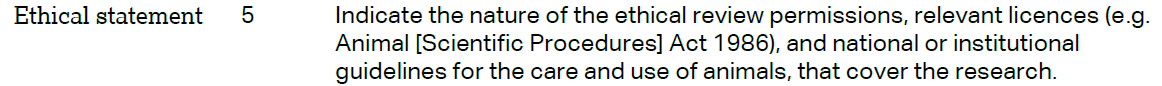 | | | The use of these animals was approved by the Committee on the Use of Live Animals in Teaching and Research of the University of Hong Kong (CULATR 2311-11), and the procedures were carried out in accordance with the institutional guidelines. The animals were kept under the standardized conditions at the Laboratory Animal Unit of The University of Hong Kong/ the Minimal Disease Area with controlled humidity-temperature environment, controlled light-dark regime (artificial light for 12 hours daily), sufficient movement allowed, free access to water and hygienic conditions were provided for the rats. |  |
| 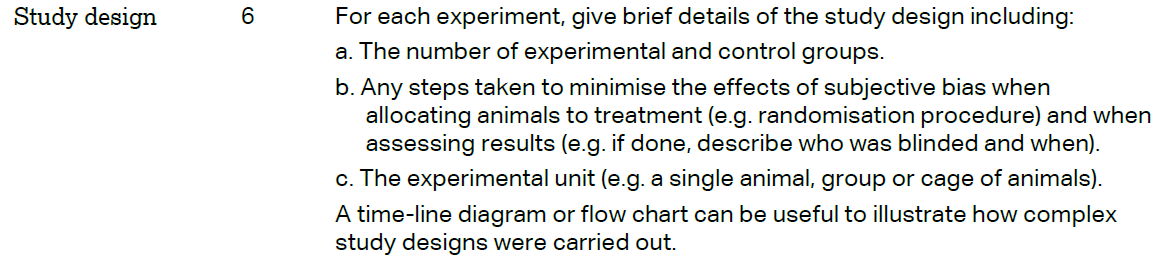 | | | Animals were sacrificed by intraperitoneal injection using 20% Dorminal (200 mg pentobarbital sodium, Alfasan, Woerden-Holland, Netherlands) with a dose of 100mg per 100g of body weight. Mandibular condyles and femoral condylar cartilage (FCC) were aseptically removed (Fig 1) to analyze two primary outcomes; histology of prepared tissue sections and RNA integrity of LCM samples. For the histological comparison of formalin-fixed paraffin embedded (FFPE) sections and unfixed frozen sections, MCCs dissected from one animal were either immediately frozen using the protocol described below, or fixed in 4% formalin, decalcified, dehydrated, embedded in paraffin, sectioned, and briefly stained with cresyl violet stain. In addition, six MCC and six FCC specimens were harvested from three rats to prepare 50 LCM samples; 10 for each MCC zone (FZ, PZ, MZ, HZ groups) and 10 samples from FCC tissue (group C) (Fig 2) Then to evaluate the integrity of LCM-RNA, qRT-PCR was performed to determine 3'/m ratio of two housekeeping genes; GAPDH and β–Actin. |  |
| 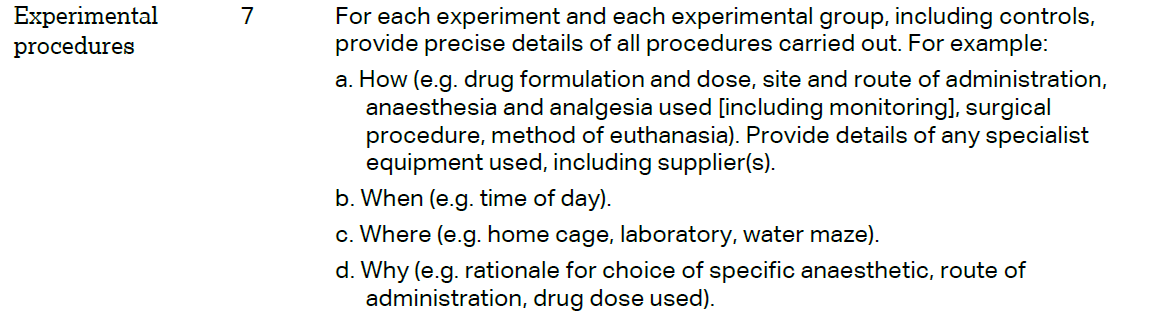 | | | The animals were kept under the standardized conditions at the Laboratory Animal Unit of The University of Hong Kong/ the Minimal Disease Area with controlled humidity-temperature environment, controlled light-dark regime (artificial light for 12 hours daily), sufficient movement allowed, free access to water and hygienic conditions were provided for the rats. Animals were sacrificed by intraperitoneal injection using 20% Dorminal (200 mg pentobarbital sodium, Alfasan, Woerden-Holland, Netherlands) with a dose of 100mg per 100g of body weight |  |
| 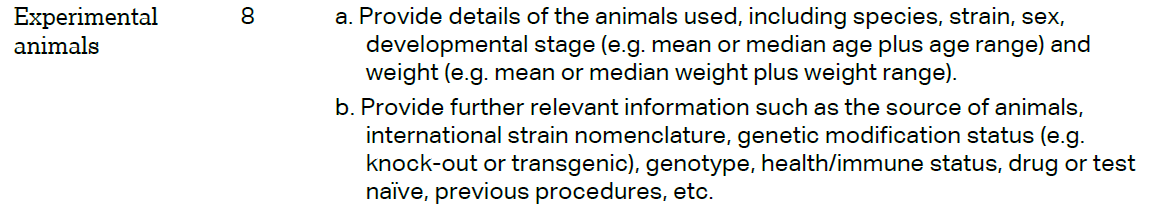 | | | As numerous properties are shared^13^, Sprague-Dawley 5-week-old male rat (Rattus norvegicus) was chosen as an experimental model. We selected the age of 5 weeks not only because MCC articulation function is already present in a more mature state, but also the maximum growth spurt for rats occurs at day 31.5^14^ |  |

The ARRIVE guidelines. Originally published in *PLoS Biology*, June 2010^1^

| 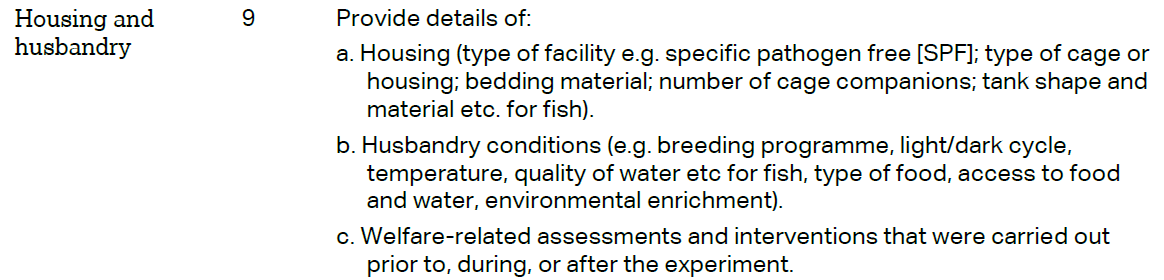 | The use of these animals was approved by the Committee on the Use of Live Animals in Teaching and Research of the University of Hong Kong (CULATR 2311-11), and the procedures were carried out in accordance with the institutional guidelines. The animals were kept under the standardized conditions at the Laboratory Animal Unit of The University of Hong Kong/ the Minimal Disease Area with controlled humidity-temperature environment, controlled light-dark regime (artificial light for 12 hours daily), sufficient movement allowed, free access to water and hygienic conditions were provided for the rats. | |
| --- | --- | --- |
| 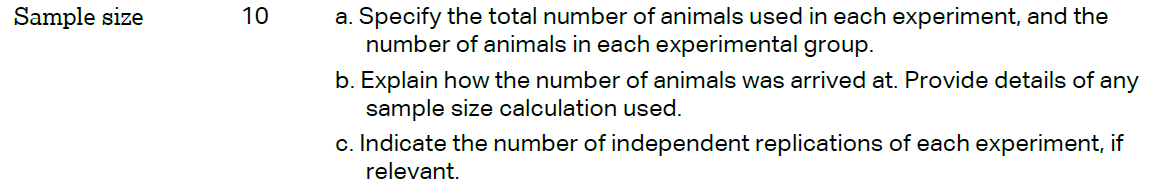 | For the histological comparison of formalin-fixed paraffin embedded (FFPE) sections and unfixed frozen sections, MCCs dissected from one animal were either immediately frozen using the protocol described below, or fixed in 4% formalin, decalcified, dehydrated, embedded in paraffin, sectioned, and briefly stained with cresyl violet stain. In addition, six MCC and six FCC specimens were harvested from three rats to prepare 50 LCM samples; 10 for each MCC zone (FZ, PZ, MZ, HZ groups) and 10 samples from FCC tissue (group C) | |
| 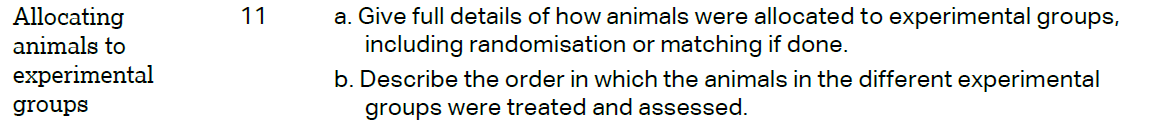 | Not applicable. | |
| 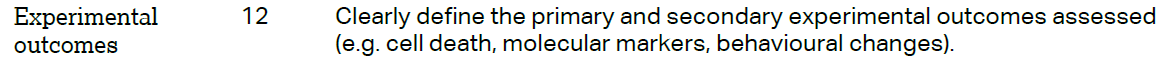 | Mandibular condyles and femoral condylar cartilage (FCC) were aseptically removed (Fig 1) to analyze two primary outcomes; histology of prepared tissue sections and RNA integrity of LCM samples. For the histological comparison of formalin-fixed paraffin embedded (FFPE) sections and unfixed frozen sections, MCCs dissected from one animal were either immediately frozen using the protocol described below, or fixed in 4% formalin, decalcified, dehydrated, embedded in paraffin, sectioned, and briefly stained with cresyl violet stain. In addition, six MCC and six FCC specimens were harvested from three rats to prepare 50 LCM samples; 10 for each MCC zone (FZ, PZ, MZ, HZ groups) and 10 samples from FCC tissue (group C) (Fig 2). Then to evaluate the integrity of LCM-RNA, qRT-PCR was performed to determine 3'/m ratio of two housekeeping genes; GAPDH and β–Actin. | |
| 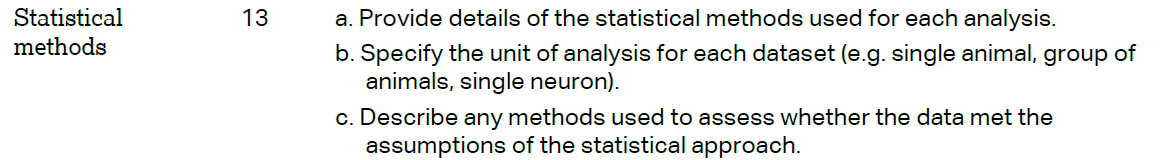 | Mandibular condyles and femoral condylar cartilage (FCC) were aseptically removed (Fig.1 a-e) to analyze two primary outcomes; histology of prepared tissue sections and RNA integrity of LCM samples. | |
| RESULTS |  | |
| 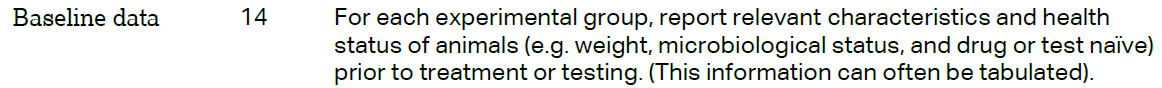 | Not applicable. | |
| 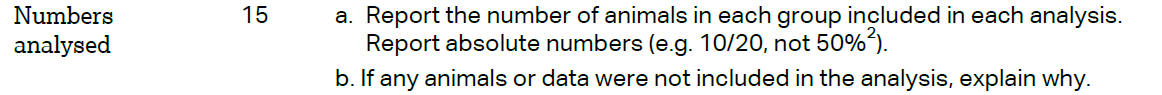 | For the histological comparison of formalin-fixed paraffin embedded (FFPE) sections and unfixed frozen sections, MCCs dissected from one animal were either immediately frozen using the protocol described below, or fixed in 4% formalin, decalcified, dehydrated, embedded in paraffin, sectioned, and briefly stained with cresyl violet stain. In addition, six MCC and six FCC specimens were harvested from three rats to prepare 50 LCM samples; 10 for each MCC zone (FZ, PZ, MZ, HZ groups) and 10 samples from FCC tissue (group C) | |
| 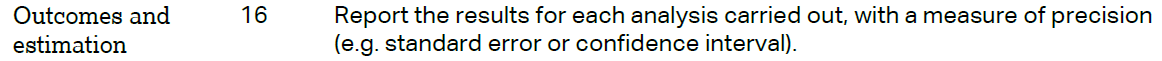 | **Table 2:** Average values of 3’/ m GAPDH and β-Actin ratio for LCM-RNA samples retrieved from groups C (the control), FZ, PZ, MZ, and HZ.   \| **GROUP** \| **3'/m GAPDH (Mean ± SD)** \| **3'/m**  **β-Actin**  **(Mean ± SD)** \| \| --- \| --- \| --- \| \| **C (femoral condylar cartilage)** \| 1.11±0.11 \| 1.41±0.24 \| \| **FZ (fibrous zone of MCC)** \| 1.28 ± 0.21 \| 1.93 ± 0.46 \| \| **PZ (proliferative zone of MCC)** \| 1.43 ± 0.23 \| 1.88 ± 0.28 \| \| **MZ (mature zone** **of MCC)** \| 1.56 ± 0.26 \| 2.12 ± 0.41 \| \| **HZ (hypertrophic zone** **of MCC)** \| 1.44 ± 0.15 \| 2.04 ± 0.29 \| | |
| 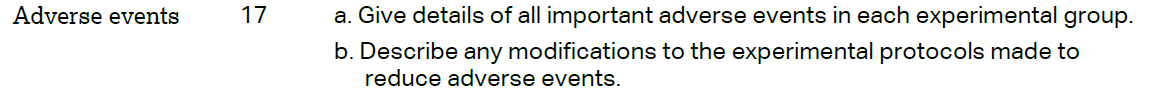 | Not applicable. | |
| DISCUSSION |  | |
| 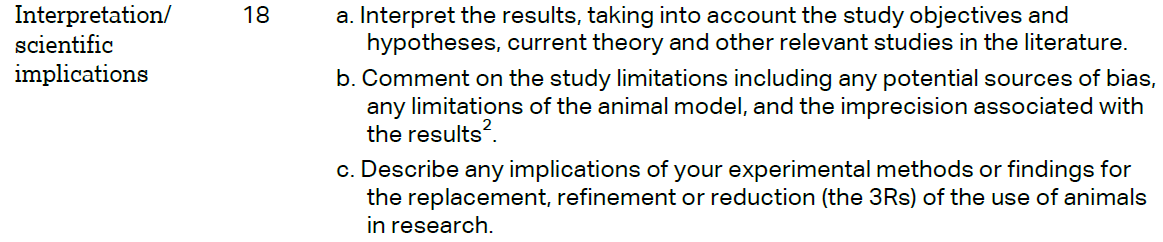 | lcm technique has been applied to a variety of tissues using different methods to prepare a wide range of biological samples. Contrary to DNA, RNA is more sensitive to specimen handling and preparation procedure, and requires strictly followed RNase-free techniques [[7](#_ENREF_7)]. Several protocols have been suggested to optimize the procedure for LCM-RNA recovery and to cope with technical challenges [[8](#_ENREF_8), [15-25](#_ENREF_15)]. One of the challenges is that LCM process lengthy and performed at room temperature. It may also require tissue staining, which exposes RNA to chemical components and aqueous solutions. Moreover, microdissecting multiple zones from the same tissue section and pooling procedures make the challenge greater [[20](#_ENREF_20)]. In addition, because of the high-cost, the LCM instrument is usually a core-facility service separately located from the histology facility. Logistic issues such as limited access time to the LCM instrument and transporting samples between facilities may further complicate the process [[7](#_ENREF_7)]. Therefore when conducting LCM experiments for RNA recovery, there are two requirements; the first is to obtain acceptable tissue section morphology that allows histological identification of the desirable cell/tissue type, and the second is to preserve the integrity and biological accessibility to RNA. Each step in the LCM protocol could have a serious impact on RNA quality, thus optimization of some crucial factors is highly important. | |
| 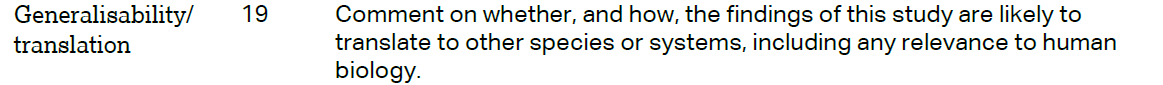 | Obtaining RNA samples with high quality, thus allowing accurate subsequent downstream analysis. Generating accurate cellular, molecular, and genetic data facilitates developing a specific molecular signature and fingerprints for a cell population or a specific pathological lesion or condition. This information, in turn, provides invaluable insights for MCC tissue-engineering field and regenerative cell-based therapy. | |
| 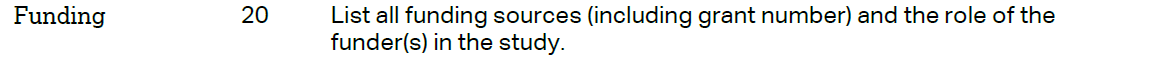 | | This study was supported by Dr. Vincent Leung sponsored funding awarded to The University of Hong Kong |


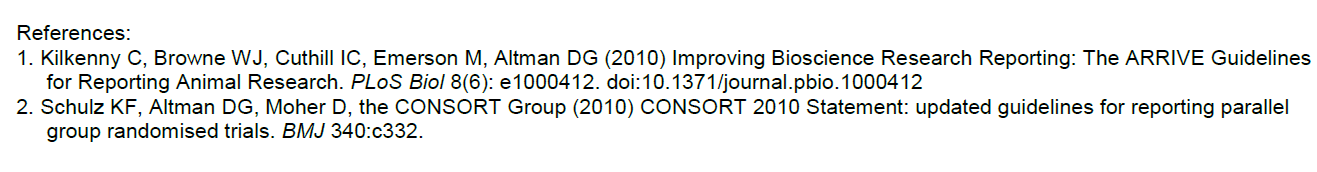

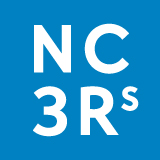


***S2 SUPPLEMENTARY FILE***

***RNASE-FREE TECHNIQUE***

To minimize exogenous RNase contamination, it is recommended to adhere to the following procedures:

- **To wear** **disposable gloves** throughout the experiment, and change them frequently.
- **Laboratory surfaces and equipment** should be decontaminated by wiping down with a commercially RNase decontamination solution, a mild solution of bleach, or NaOH, followed by wiping with absolute ethanol, and then rinsing twice with nuclease-free water.
- **Glassware and metal ware** are either decontaminated as above or baked at 450ºF (230ºC) for two hours or more.
- **Plastic ware** should be certified as RNase-free item upon purchasing (such as RNase-free tubes and pipette tips). If used more than one time (for instance slide boxes, slide chambers, as well as staining and dehydration plastic jars), it is rinsed with absolute ethanol, followed by distilled water, then treated with the commercial RNase decontamination solution, rinsed again with nuclease-free water, and finally allowed to dry in a hood.
- **Chemical reagents and solutions** are either certified as RNase-free by the manufacturer, treated with diethylpyrocarbonate (DEPC). In addition, RNase inhibitor, for example SUPERase In™ RNase Inhibitor, ProtectRNA RNase Inhibitor, or RNasin® Plus RNase Inhibitor, could be added to the used solutions. All solutions should not be reused or poured back into their containers.
- **Slides** are dipped into the commercial RNase decontamination solution for few seconds, and then washed two times separately with nuclease-free water, dried for two hours at 37-55ºC, followed by UV-treatment shortly before use to destruct any potentially contaminating RNases.
